# Virgin Coconut Oil as a New Concept for Periodontal Tissue Regeneration via Expressions of TNF-*α* and TGF-*β*1

**DOI:** 10.1155/2022/7562608

**Published:** 2022-02-08

**Authors:** Hasanuddin Thahir, Arni Irawaty Djais, Mansjur Nasir, Ayu Rahayu Feblina, Afdalia Annisa, Nir Etriyani, Harun Achmad

**Affiliations:** ^1^Department of Periodontics, Faculty of Dentistry, Hasanuddin University, Makassar, Indonesia; ^2^Department of Orthodontic, Faculty of Dentistry, Hasanuddin University, Makassar, Indonesia; ^3^Periodontology Specialist Dental Educational Program, Faculty of Dentistry, Hasanuddin University, Makassar, Indonesia; ^4^Department of Pediatric Dentistry, Faculty of Dentistry, Hasanuddin University, Makassar, Indonesia

## Abstract

**Background:**

Virgin coconut oil is a natural product from coconut that has many benefits such as antibacterial, anti-inflammatory, and antioxidant. Inflammatory disease of the periodontal tissues has a high prevalence worldwide. The main etiology of periodontitis plaque of biofilm contains colonies of pathogenic microorganisms. The occurrence of inflammation in the periodontal tissue stimulates the release of inflammatory mediators, such as TNF-*α* and TGF-*β*. Treatment for periodontitis can be performed starting from initial therapy and usually accompanied by additional therapy such as local drug delivery. VCO can be used as an alternative to antibiotics.

**Objective:**

To determine the effectiveness of VCO gel on periodontal tissue regeneration materials through the expression of TNF-*α* and TGF-*β*1.

**Methods:**

This is an experimental laboratory with a posttest-only control group design. VCO was made from grated fresh coconut and then mixed with NaCMC to obtain gel viscosity. The subjects of this study were 30 male periodontitis-induced Wistar rats by injecting *Porphyromonas gingivalis* into the gingival sulcus. Wistar rats were then divided into 3 groups. On the 7th and 14th days, the rats were sacrificed and the jaw was sampled to determine the amount of TNF-*α* and TGF-*β*1 expression in the regenerative process of periodontal tissue.

**Result:**

The amount of TNF-*α* and TGF-*β*1 increased significantly in the treatment group, but not as much as the increase in the positive control and negative control groups.

**Conclusion:**

VCO gel can affect the expression of TNF-*α* and TGF-*β*1 in the regeneration process of periodontal tissue in periodontitis-induced rats.

## 1. Introduction

Increasing public awareness in utilizing natural ingredients as medicines has led to an increasingly rapid increase in the production of medicinal plants. This is because drugs derived from natural ingredients have relatively small side effects [1]. Coconut plant is one of the natural products in which almost all of its parts can be utilized, both for food and beverage ingredients, as well as those processed into virgin coconut oil (VCO). VCO oil can be obtained from fresh coconut flesh or from copra. The process of making VCO can be carried out in 2 ways, namely, VCO from fresh coconut flesh, known as the wet process because in this process, water is added to extract oil, while the manufacture of VCO with raw copra is known as the dry process [2–4]. The heating process in the manufacture of VCO produces compounds containing lauric acid, so they have antibacterial properties [5, 6].

VCO contains saturated fatty acids including Medium-Chain Fatty Acids (MCFAs) and Medium-Chain Triglycerides (MCTs). MCFA is a lauric acid which has antiprotozoal, antiviral, and antibacterial properties. MCT in VCO can increase immunity against disease and accelerate healing from illness and can prevent obesity [1, 7, 8]. There are many studies that use VCO as a treatment for various diseases. In 2017, a study found the effect of VCO on *A. actinomycetemcomitans* and *P. gingivalis* which are the main bacteria that cause periodontal disease [7].

Periodontal disease is a disease caused by damage to the supporting tissues of the teeth that starts with reversible inflammation of the gingiva, which then gets worse until bone destruction occurs known as periodontitis. According to the FDI World Dental Federation in 2015, several countries in the world in 2010 had high prevalence of periodontitis, including Australia with a prevalence of more than 15%, while in Indonesia, almost all regions had a prevalence of more than 15%. The prevalence of periodontitis in people aged 15 years according to Riskesdas 2018 data is 67.8%, and this means that, out of ten people in Indonesia, 7 people suffer periodontitis [9, 10].

The etiology of periodontal disease can be divided into two groups, local factors and systemic factors. Local factors are usually caused by bacterial plaque, especially *Porpyromonas gingivalis (P. gingivalis), Tanerella forsythia, Provotella intermedia,* and *Treponema denticola* which are often found in destructive periodontitis with deep pockets. One of the systemic is synthetic hormones found in hormonal contraceptives containing progesterone and estrogen [11–13]. Periodontitis is a multifactorial disease, with interactions between microbial infection and host response, in which microbial biofilms are considered the main etiologic agent to initiate inflammation, resulting in loss of periodontal attachment, increased pocket depth, and loss of alveolar bone [14, 15].

When inflammation occurs in the body, the immune response will release cytokines, chemical messengers, or intermediaries in intercellular communication. These inflammatory cytokines include TNF-*α*, IL-1*β*, IL-6, IL-8, and superoxide anion in human monocyte cell culture. Tumor Necrosis Factor-*α* (TNF-*α*) is a cytokine that stimulates cell proliferation and differentiation. Cardoso et al. [12, 16] derived from neutrophils, monocytes, and macrophages. One of the possible infection mechanisms is activation that stimulates monocytes and neutrophils to produce TNF-*α*, which causes tissue disruption. Increased levels of TNF-*α*, which are part of the pathogenesis of infection through the mechanism of phagocytic cells, such as polymorphonuclear, neutrophil, monocytes, and macrophages, will trigger the release of chemical mediators such as TNF-*α* that play a role in the occurrence of periodontal disease [17–19]. Neto et al. in 2018 found in their study there is a balanced between the expression of TNF-*α* as proinflammatory cytokines in periodontal disease caused by a periapical lesion and TGF-*β* as a anti-inflammatory cytokines after endodontic treatment which shows that two cytokines play a role in the periodontal regeneration process [20].

Growth factors regulate growth and differentiation of cells [21]. Transforming growth factors (TGFs) are divided into TGF-*α* and TGF-*β*, which are synthesized by various normal cells and platelets. They affect the activation of macrophages, fibroblast proliferation, synthesis of connective tissue fibers and their matrix, local angiogenesis, healing, and also, regulation of lymphocytes [22–24]. TGF-*β*1 is synthesized by various normal cells and platelets and affects macrophage activation, fibroblast proliferation, synthesis of connective tissue fibers and their matrix, local angiogenesis, healing, and regulation of T lymphocytes [25, 26]. TGF-*β*1 plays a very important role in the inflammatory response. The aim of this study was to determine the effectiveness of VCO gel on the expression of TNF-*α* and TGF-*β*1 in the tissue regeneration process after periodontitis.

## 2. Methods

This is an experimental laboratory with a posttest-only control group design. VCO was made from grated fresh coconut and left for up to 24 hours to obtain colorless and odorless pure coconut oil. Then, the VCO was mixed with NaCMC to form gel for easy application during the research treatment. The subjects of this study were male Wistar rats weighing 150–200 grams which were induced by periodontitis by inserting a silk ligature on the lower anterior teeth and injecting P*. gingivalis* ATCC 33277 bacteria which were prepared in per liter culture media: 5 g yeast extract, 5 g peptone, and 200 ml goat blood filled up to 1 liter. Then, it was autoclaved for 15 minutes and put into the gingival sulcus of Wistar rats. After 5 days, periodontitis will form on the gingiva of Wistar rats which is characterized by a change in the colour of the gingiva and an increase in pocket depth.

Wistar rats were divided into 3 groups, the treatment group receiving initial SRP therapy and VCO gel, the positive control group receiving SRP and metronidazole gel, and the negative control group only receiving SRP. Furthermore, euthanasia was carried out on the 7th and 14th days after treatment, with 5 Wistar rats from each treatment group. Then, the Wistar rats were fixed on the workbench for decapitation and separation of the cranium from the mandible. Each jaw piece was fixed in 10% buffered formalin, which would then be made into HPA preparations. The expression of TNF-*α* and TGF-*β*1 was then determined by immunohistochemical staining using TNF-*α* and TGF-*β*1 antibodies and viewed under a light microscope with 1000x magnification for further calculation of the number of expressions. The data obtained were tested for normality using the Kolmogorov–Smirnov test, and then, the data were tested for homogeneity using Levene's test. To analyze the differences between the research groups, one-way ANOVA was used. The results of the analysis were declared significant or having the difference if the *p* value <0.05.

## 3. Results

On the fifth day after the binding of silk ligature and administration of *P. gingivalis* bacteria into the mandibular anterior gingival sulcus, periodontitis occurred in Wistar rats, characterized by changes in gingival colour and increased pocket depth. SRP as initial therapy for periodontitis was combined with VCO gel in the first group, SRP was combined with metronidazole gel in the positive control group, and only SRP was performed in the negative control group. The histopathological results in all research groups showed lymphocyte cells expressing TNF-*α* and TGF-*β*1 in the mandibular gingival sulcus that had been induced by periodontitis.

### 3.1. TNF-*α* Expression

The results of immunohistochemical examination, TNF-*α* expression, were observed on the 7th and 14th days in the three treatment groups.  Table 1 shows the amount of TNF-*α* expression in male Wistar rats with periodontitis. In the treatment group with initial SRP therapy combined with VCO gel and the negative control group with SRP treatment alone, a significant increase was seen in the amount of TNF-*α* expression (*p* < 0.05) between day 7 and day 14, while in the positive control group, the combination of SRP and metronidazole gel showed the amount of TNF-*α* expression on the 7th and 14th days was not significant (*p* > 0.05).

**Figure 1:**
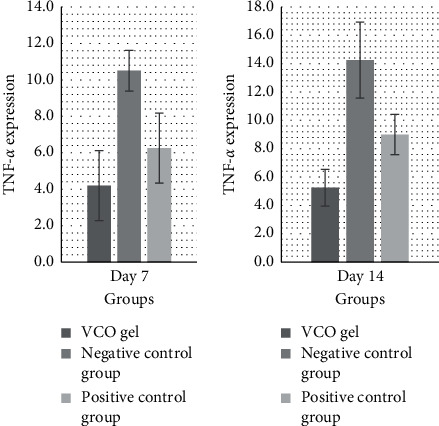
Bar charts indicating TNF-*α* expression on the 7th and 14th day.

The bar chart in Figure 1 shows an increase in TNF-*α* expression in the three groups, on the 7th and 14th day. On the 7^th^ day, TNF-*α* expression in all groups increased, indicating an inflammatory response due to periodontitis. Combination of SRP and VCO gel showed an increase in TNF-*α* expression that was not as high as that of the positive control group and the negative control group on the 14th day, which indicated that VCO gel administration could suppress inflammation.

**Figure 2:**
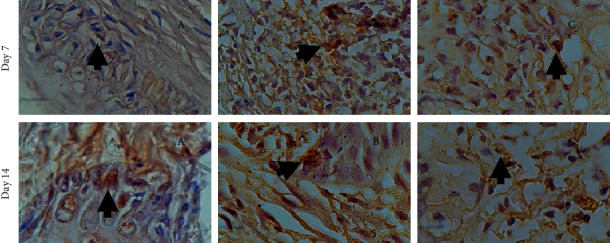
TNF-*α* expression on the 7th and 14th day. (a) Treatment group: combination of SRP + VCO gel. (b) Positive control group: SRP + metronidazole gel. (c) Negative control group: SRP.

**Figure 3:**
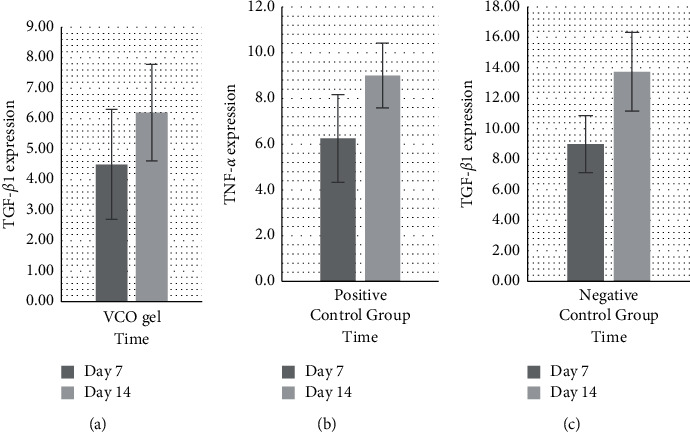
(a) Bar chart showing increased TNF-*α* on the 7th and 14th day from the three groups. On the 7th day, the group in combination of SRP dan VCO gel had a significant increase. The increase was not as high as in the (b) positive control and (c) negative control group.

Immunohistochemical examination of TNF-*α* expression (Figure 2) performed by immunohistochemical staining and TNF-*α* antibody (1000x magnification) showed an increased TNF-*α* expression indicated by brownish colour on the 7th and 14th days after treatment (*p* < 0.05). These results are shown as a bar chart in Figure 3.

### 3.2. TGF-*β*1 Expression

Table 2 shows the amount of TGF-*β*1 expression on the 7th and 14th days. It can be seen that all groups, the treatment group, positive control, and negative control, experienced a significant increase in TGF-*β*1 expression between the 7th and 14th days.

**Figure 4:**
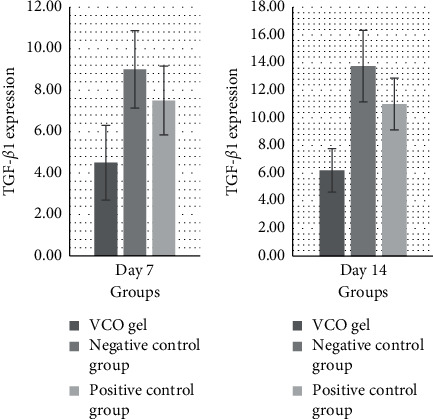
Chart bars showing TNF-*α* expression from the three groups on the 7th and 14th day.

**Figure 5:**
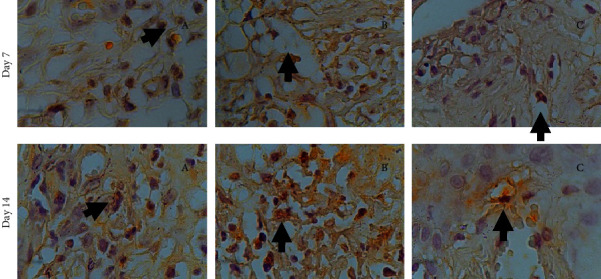
TGF-*β*1 (brown) in immunohistochemistry preparation on the 7th and 14th day. (a) Treatment group: combination of SRP + VCO gel. (b) Positive control group: SRP + metronidazole gel. (c) Negative control group: SRP.

**Figure 6:**
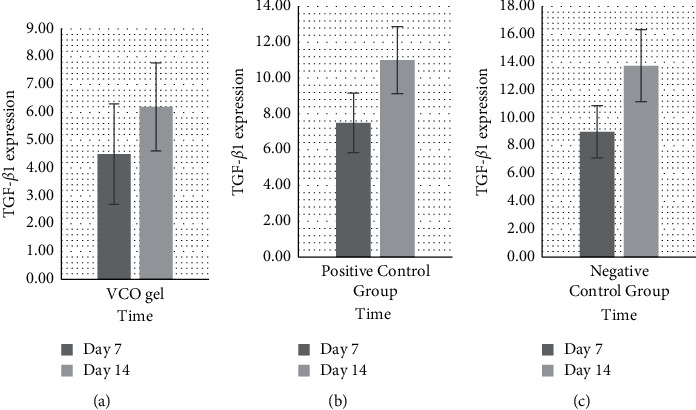
The increase in TGF-*β*1 on the 7th and 14th day in the three groups. (a) The SRP and VCO gel combination treatment group has small increase. (b) The group that received VCO gel and metronidazole gel has higher increase, as well as (c) the negative control group with SRP alone.

Based on the bar chart (Figure 4), from the average increase in TGF-*β*1 expression in the three groups, it can be seen that the treatment group experienced a significant increase amount of TGF-*β*1 (*p* < 0.05), compared to the other groups. The increasing amount of TGF-*β*1 expression was also smaller when compared to the that of positive control group and the negative control group on the 7th and 14th day.  Figure 5 shows the immunohistochemical staining and TGF-*β*1 antibody (1000x magnification). The increase of TGF-*β*1 expression on the 7th and 14th day in the three groups is shown in bar charts in Figure 6.

## 4. Discussion

Periodontitis is defined as an inflammatory disease of the tooth-supporting tissue, caused by certain microorganisms. In this study, *P. gingivalis* bacteria were induced in the gingiva of Wistar rats, where the major causative agent in periodontitis is this bacterium which has been identified by the researchers and is associated with the severity of periodontal disease [27]. The treatment group with the administration of VCO gel in periodontitis-induced Wistar rats experienced a significant increase in TNF-*α* expression, compared to other groups.

Periodontitis TNF-*α* is a proinflammatory cytokine whose sources are neutrophils, monocytes, and macrophages. One of the possible infection mechanisms is activation that stimulates monocytes and neutrophils to produce TNF-*α*, which causes tissue disruption. Increased levels of TNF-*α* are part of the pathogenesis of infection through the mechanism of phagocytic cells, as polymorphonuclear, neutrophil, monocytes, and macrophages will trigger the release of chemical mediators such as TNF-*α* cytokines that play a role in periodontal disease [28–30].

An increase in TNF-*α* will upregulate the receptor activator of RANKL NF-*κβ* ligand (RANKL). The upregulation of TNF-*α* and RANKL will increase the production of receptor activator of RANKL NF-*κβ* (RANK). This results in a binding between RANK and RANKL which then causes the uptake of the TNF-Receptor Associated Factor-6 (TRAF-6) adapter protein. TRAF-6 will induce *c* Fos and activator protein 1 (AP-1), which in turn induces Nuclear Factor of Activated T cells c1 (NFAT C1). RANK-RANKL binding that causes the uptake of the adapter protein TNF-Receptor Associated Factor-6 (TRAF-6) will cause a signal transduction sequence that causes increased activation of NFATC1, which functions as a transcription factor for osteoclast formation and enhances the number of mature osteoclasts. These mature osteoclasts then form active osteoclasts, which stimulate periapical alveolar bone resorption [17, 30, 31].

Cytokine production is an immune response. The expression of TNF-*α* and TGF-*β*1 on the 7th day increased in small amounts on the 14th day when the repair phase had begun. TNF-*α* and TGF-*β*1 increased significantly when compared with those of the positive control group and the negative control group. The significant increase in TNF-*α* expression (*p* < 0.05) in this study was probably due to the lauric acid content in the VCO gel which functions in the inflammatory process and also has antibacterial properties, so it can accelerate the tissue regeneration and healing process.

In this study, it was proven that there was a significant increase in the number of TNF-*α*-expressing cells in periodontal tissues that were inflamed due to the induction of *P. gingivalis* bacteria by administration of VCO gel, not as much as TNF-*α* expression in the positive control group and negative control group. This proves that the VCO gel helps in suppressing the inflammatory process so that it can accelerate tissue regeneration.

It was also found that the number of TGF-*β*1-expressing cells in the periodontal tissue that experienced inflammation due to the induction of *P. gingivalis* bacteria was higher than that in the periapical tissue in the negative control group but lower when compared to that in the positive control group considering that TGF-*β*1 is a multifunctional regulator of cell growth and differentiation during formation and repair. Moreover, TGF-*β*1 has various (pleiotropic) effects, including as a growth inhibitor for most epithelial cells and for leukocytes. Since inflammation is still occurring in the periodontal tissue, the release of TGF-*β*1 is blocked.

This research conducted by Nevin et al. on 18 Sprague Dawley with excision wounds proved that VCO was able to increase the proliferation of fibroblast cells so that the density of collagen fibers increased, whereas wounds treated with VCO healed faster, indicated by a decrease in complete epithelialization time and a higher rate of reepithelialization of various skin components. Pepsin-soluble collagen also showed a significant increase in VCO-treated wounds [32, 33]. Another study conducted by Jannah et al. on *Rattus norvegicus* applied VCO gel after tooth extraction. The results obtained were an increase in the number of fibroblasts 0.4 times that in the group that was applied to VCO compared to povidone iodine. VCO also affects dermal and epidermal healing and provides strength to epithelial tissue [32].

The VCO gel which is produced from coconut flesh is known to have the ability to accelerate cell metabolism and has anti-inflammatory and anti-infection properties, with minimal possibility of resistance to chemical medicament. Further research with various concentrations of VCO is needed to determine the best levels in the therapy tissue regeneration.

## 5. Conclusions

Based on the results of this study, it can be concluded that there was a significant increase in the expression of TNF-*α* and TGF-*β*1 in periodontitis-induced Wistar rats with SRP treatment and VCO gel administration, which is a marker of periodontal tissue regeneration. Further studies are needed with a larger study population to analyze the VCO gel on periodontal regeneration, both in soft and hard tissues, with various levels of VCO concentrations.

## Figures and Tables

**Table 1:** Mean of TNF-*α* expression on the 7th and 14th day in each treatment group.

Group	Sample	7th day	14th day	p
		(Mean ± SD)	(Mean ± SD)	
Treatment (SRP + VCO gel)	5	4.25 ± 1.920	5.25 ± 1.299	^*∗*^0.048
Positive control (SRP + metronidazole gel)	5	6.25 ± 1.920	9.00 ± 1.414	0.192
Negative control (SRP)	5	10.50 ± 1.118	14.25 ± 2.681	^*∗*^0.032

**Table 2:** Mean of TGF-*β*1 on the 7th and 14th day in the respective treatment group.

Group	Sample	7th day	14th day	p
		(Mean ± SD)	(Mean ± SD)	
Treatment (SRP + VCO gel)	5	4.50 ± 1.803	6.20 ± 1.581	^*∗*^0.008
Positive control (SRP + metronidazole gel)	5	7.50 ± 1.658	11.00 ± 1.871	0.078
Negative control (SRP)	5	9.00 ± 1.871	13.75 ± 2.586	0.078

## Data Availability

The data sets generated and/or analyzed during the current study are available from the corresponding author on reasonable request.
